# Analysis of Disparities in Diagnosis of Autism Spectrum Disorder in the Military Health System Pediatrics Population

**DOI:** 10.1007/s10803-024-06703-w

**Published:** 2025-01-09

**Authors:** Ocheze Chikezie-Darron, Joshua Sakai, Daniel Tolson

**Affiliations:** 1 Department of Pediatrics, Madigan Army Medical Center, Tacoma, WA USA; 2 Department of Clinical Investigation: Data Science Research Service, Madigan Army Medical Center, Tacoma, WA USA

**Keywords:** Autism, Autism spectrum disorder, Diagnosis, Disparities, Military

## Abstract

There have been disparities reported in prevalence of autism by gender, race, and socioeconomic status with older ages of diagnosis in non-White and in female children. Possible disparities in the ages of autism diagnosis are not well-established within the Military Health System (MHS) pediatric population, where we hypothesized less disparities given universal Tricare coverage for active-duty military families and theoretically equal access to the military treatment facility (MTF). We conducted retrospective cross-sectional analysis using deidentified database repository records from the MHS. We collected and analyzed demographic data on children covered by Tricare and newly diagnosed with autism within an MTF (N = 31,355) or outside of the MTF (5,579 respectively). Within the MTF, we identified younger ages of autism diagnosis in non-White children less than 18 years old (p < 2.2e^−16^), without significant differences in ages of diagnosis by race in children less than 6 years of age. There were no statistically significant differences in ages of diagnosis between males and females. Outside the MTF, we identified younger ages of autism diagnosis in males versus females with statistically significant difference in average ages of autism diagnosis between males and females less than the age of 18 years (p = 4.4e-08). This difference was not seen in children less than 6 years of age. Racial data was not available for diagnosis outside the MTF. The age of autism diagnosis in the military pediatric population within the MTF did not reflect historical disparities seen in non-White and in female children.

Autism spectrum disorder, a neurodevelopmental condition, impacts a person’s social communication and behaviors. The incidence and prevalence of autism continues to rise, with latest prevalence reported by the CDC’s Autism and Developmental Disabilities Monitoring (ADDM) Network to be 1 in 36 children (Data & Statistics on Autism Spectrum Disorder, 2023). Early recognition, diagnosis, and establishment of supportive therapy for autism spectrum disorder can optimize outcomes for individuals who require support. Delays in diagnosis can lead to delays in implementing early interventions or needed supports. There have been disparities reported in the prevalence of autism by gender, race, and socioeconomic status (Rosenberg et al., 2011; Mandell et al., 2010; Durkin et al., 2017). While recent studies have shown lessening disparities in the prevalence rates of autism in Black and in White children at the ages of 4 and 8 years (Maenner et al., 2021; Shaw et al., 2021), there continues to be concern of possible underdiagnosis of autism in non-White children whose presentation are not severe. Black children have been reported to disproportionately have more deficits and lower cognitive scores at presentation compared to White children (Maenner et al., 2021; Habayeb et al., 2022; Fombonne & Zuckerman, 2022). Historically higher average age of diagnosis of non-White children (Wiggins et al., 2020) raises concern for possible underdiagnoses of autism in these children and highlights possible ongoing issues with access to care. The factors that influence or drive these disparities are not fully understood and likely involve multiple complex intertwined variables such as access to care issues (Aylward et al., 2021), parental education and cultural factors (Donohue et al., 2017), as well as possible provider bias (Constantino et al., 2020; Broder-Fingert et al., 2020).

Past studies examining whether general disparities exist in the military healthcare system have shown varying results, with some showing a lack of disparities while others show the presence of disparities but to a lesser degree than in the civilian population (Klin et al., 2015; Forester et al., 2008; Koehlmoos et al., 2021; Chaudhary et al., 2019). Knowledge of possible disparities in the diagnosis of autism within the military pediatric population is not well-established. Past studies regarding autism spectrum disorder have focused largely on other issues like challenges faced by military families with children diagnosed with autism, systematic ways to address unique needs with caring for a child with autism in the military system (Hammon et al., 2023; Aronson et al., 2016), and the association of autism diagnosis with other co-occurring conditions (Lozada, et al., 2015; Lee et al., 2018). We therefore sought to evaluate for disparities in autism diagnosis within the military population. We hypothesized that within the Military Health System (MHS) pediatric population, there would not be previous disparities of older ages of autism diagnosis in non-white children or in female children given the universal coverage of Tricare and the availability of theoretically equal access to the military treatment facility (MTF).

## Methods

We conducted a retrospective cross-sectional analysis using deidentified records from the Military Health System database to ascertain the age of autism diagnosis of the military pediatric population. We collected data (age, race, gender) on children covered by Tricare who were diagnosed with autism at an MTF or outside of the MTF at civilian/community facilities (N = 31,355 and 5579 respectively) from January 2018 to April 2023 (Table 1). Autism spectrum disorder diagnosis was defined as having a visit encounter with an ICD-10-CM diagnostic code for autism or autism spectrum disorders. We included military children with a new diagnosis of autism or autism spectrum disorder before the age of 18 years. We excluded individuals with initial visit encounters that had a diagnostic code of autism or autism spectrum disorder to prevent capturing those entering the military system with an already established diagnosis of autism or autism spectrum disorder. We performed descriptive analysis on the ages of new diagnosis and analyzed available demographic data to compare these based on gender and race. We conducted a Kruskal–Wallis for Race and Mann–Whitney U for Sex to evaluate for possible statistical significance. We chose the Kruskal–Wallis test, a nonparametric test to determine if there are statistically significant differences in age of autism diagnosis between our categorical groups (various racial groups) as it does not assume normality of data and is less sensitive to outliers than the one-way ANOVA. The Mann–Whitney U test, while similar to the Kruskal–Wallis test, is used for the comparison of two independent groups. We used it to determine if there are statistically significant differences in age of autism diagnosis between males and females. Given that past studies have noted average age of autism diagnosis to be 4 years, with average age of about 5 years in African American children (Constantino et al., 2020), we further analyzed a subgroup of less than 6 year of age to investigate possible racial or gender disparities in average age to autism diagnosis in this subgroup within the military health system.

**Table 1:** Demographics of Tricare-covered children newly diagnosed with autism from January 2018 to April 2023

Variable	MTF (N = 31,355)	Non-MTF (N = 5579)
Gender		
Male (%)	23,907 (76)	3979 (71)
Age range (years)		
0–6 (% of males)	10,784 (45)	639 (16)
7–18 (% of males)	13,123 (55)	3,340 (84)
Female (%)	7448 (24)	1600 (29)
Age range (years)		
0–6 (% of females)	3,444 (46)	218 (14)
7–18 (% of females)	4,004 (54)	1382 (86)
Race		
Black (%)	1,399 (4)	–
White (%)	4,663 (15)	–
American Indian or Alaskan Native (%)	10 (< 1)	–
Asian or Pacific Islander (%)	309 (1)	–
Other (%)	3,786 (12)	–
Unknown (%)	21,188 (68)	–

*MTF* military treatment facility, Racial data was not available in the study database for Tricare-covered children seen outside the MTF

## Results

### Within the Military Treatment Facility

**Fig. 1:**
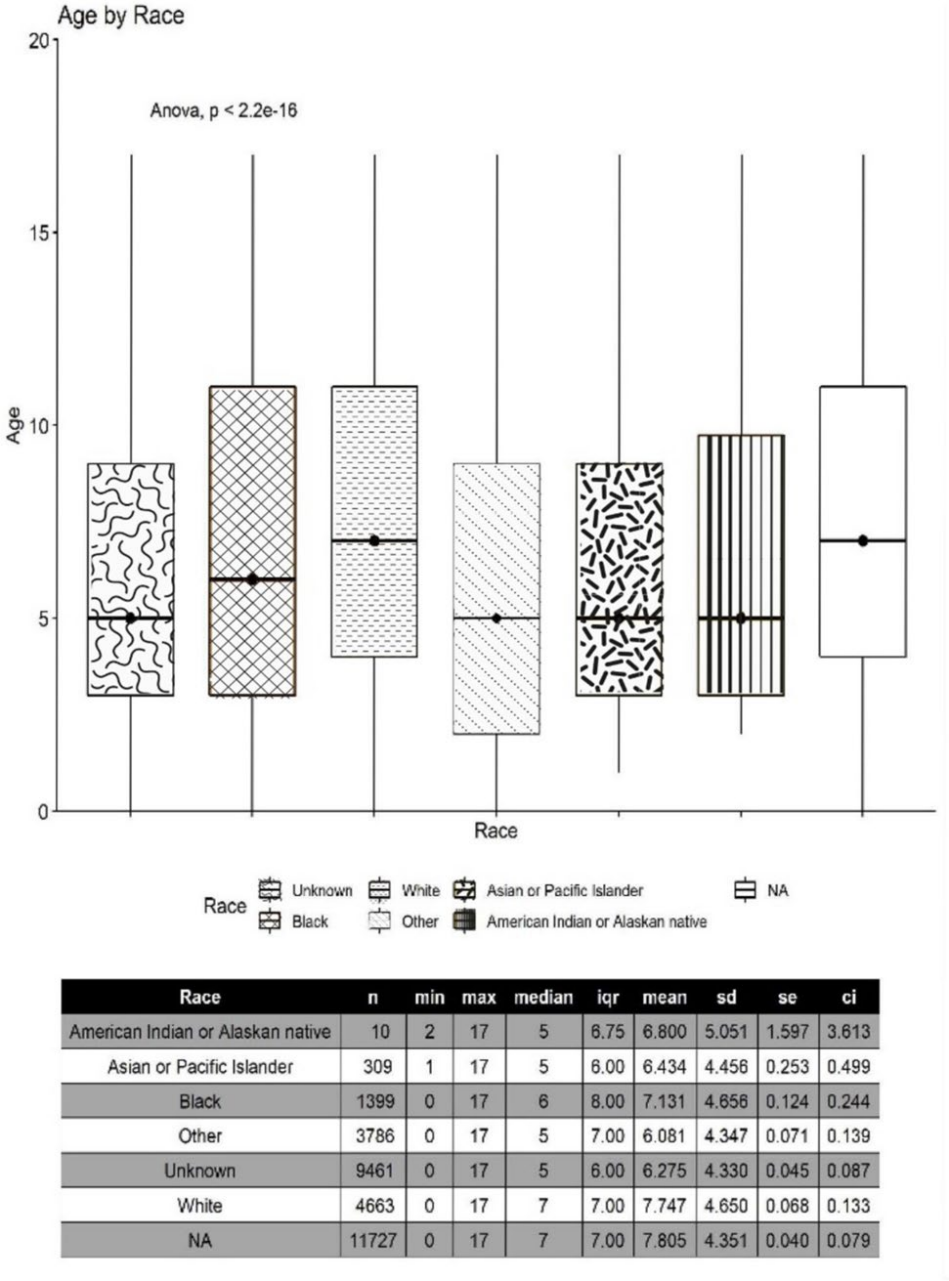
Average age of autism diagnosis by race within the MTF for the < 18 year population. Racial data was not available in the study database for Tricare-covered children seen outside the MTF

**Fig. 2:**
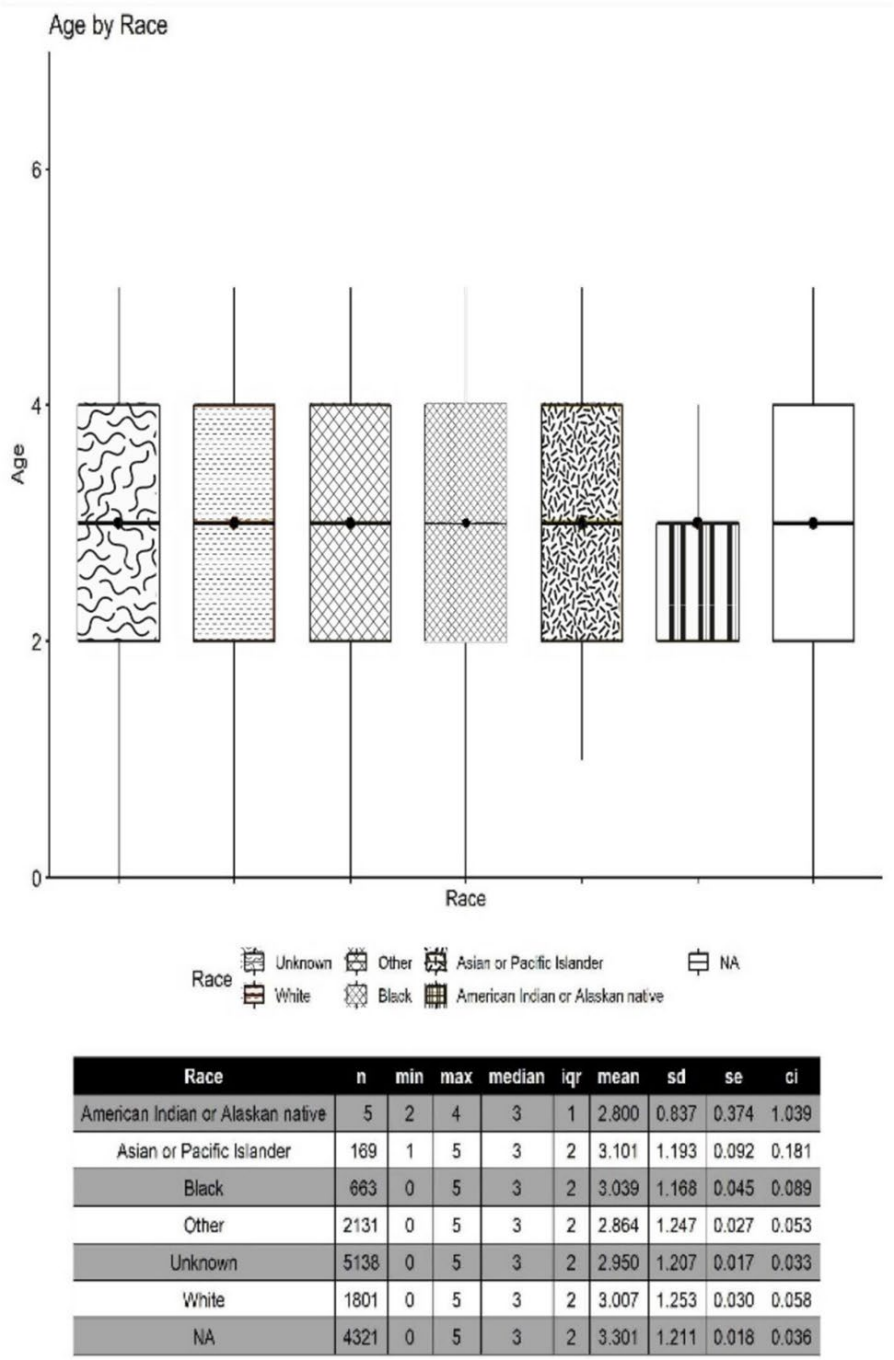
Average age of autism diagnosis per race within the MTF for the < 6 year population. Racial data was not available in the study database for Tricare-covered children seen outside the MTF

**Fig. 3:**
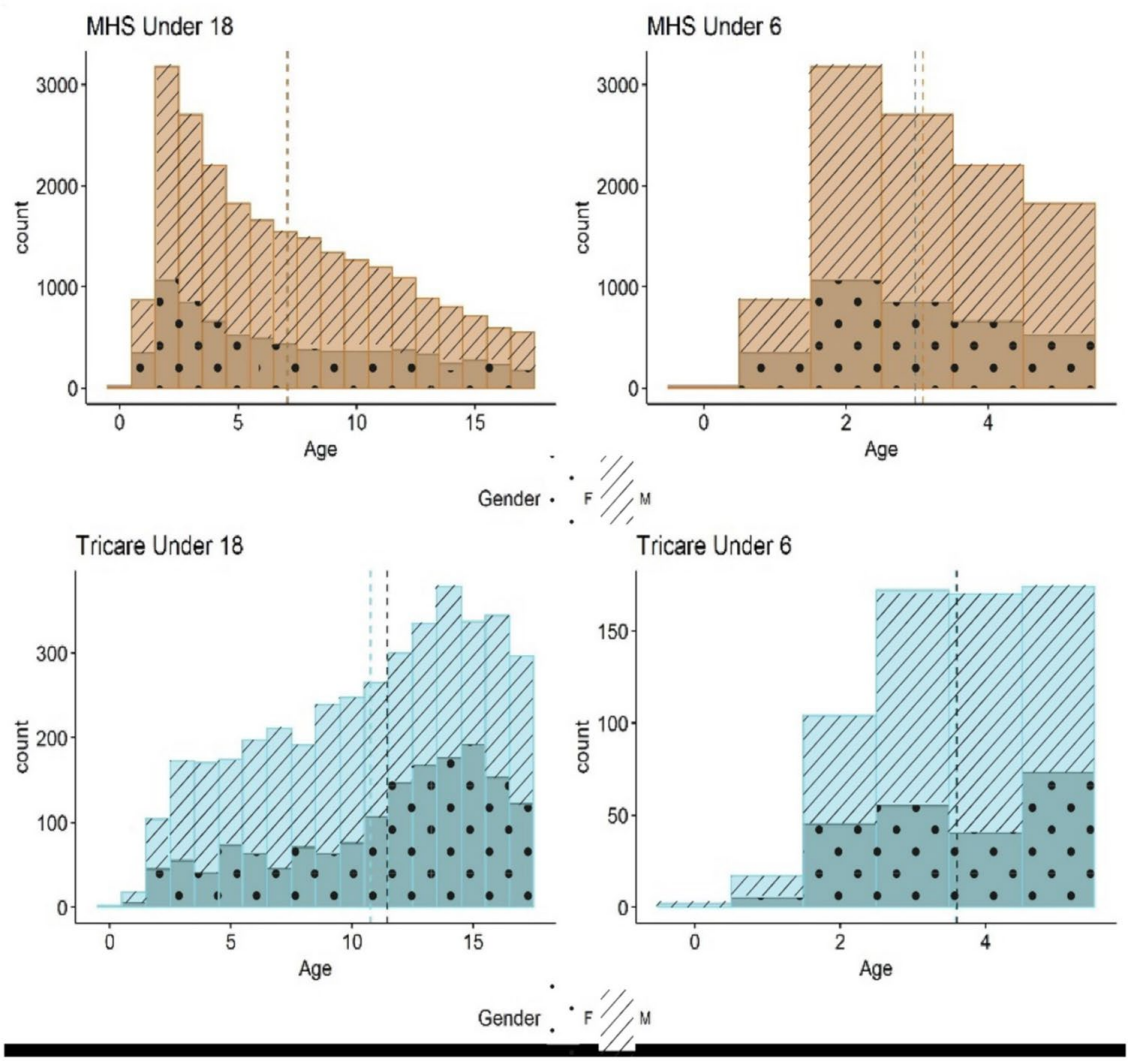
Ages of autism diagnosis by gender in the MTF (MHS) and in the community (Tricare) for patients < 18 years of age and < 6 years of age

We identified statistically significant differences between ages of autism diagnosis by race (p < 2.2e^−16^) in our study population of less than 18 year-olds, whereby non-White children (Black, American Indian or Alaskan Native, Asian or Pacific Islander) were noted to have younger average ages of autism diagnosis than White children (Fig. 1). This was also the case in other groups with unidentified racial identities (other, unknown) except for the N/A group which had a slightly higher age of new autism diagnosis (7.8 years) when compared to the age of autism diagnosis in White children (7.7 years). We did not identify significant differences in ages of autism diagnosis by race in those less than the age of 6 years (Fig. 2). There were no significant differences in the ages of autism diagnosis between males and females diagnosed within the MTF (Fig. 3). Both males and females less than the age of 18 years had an average autism diagnosis age of 7.1 years (Table 2). No significant difference was noted in the ages of autism diagnosis in children less than the age of 6 years within the MTF. Males less than the age of 6 years had an average autism diagnosis age of 3.1 years. Females less than the age of 6 years had an average autism diagnosis age of 3.0 years (Table 2).

**Table 2:** Average age in years of autism diagnosis for children with Tricare coverage diagnosed within Military Treatment Facilities (MTF) compared with the average age of autism diagnosis for children with Tricare coverage and diagnosed outside the MTF in Tricare-accepting community facilities

Tricare (outside the MTF) ages < 18y									
Variable	n	Min age	Max age	Median	iqr	Mean	SD	SE	CI
Gender									
F	1600	0	17	13	6	11.466	4.249	0.106	0.208
M	3979	0	17	12	7	10.77	4.39	0.07	0.136

MTF ages < 18y									
	n	Min age	Max age	Median	iqr	Mean	SD	SE	CI
Gender									
F	7448	0	17	6	8	7.11	4.626	0.054	0.105
M	23,907	0	17	6	7	7.074	4.421	0.029	0.056

Tricare (outside the MTF) ages < 6y									
	n	Min age	Max age	Median	iqr	Mean	SD	SE	CI
Gender									
F	218	0	5	4	2	3.601	1.211	0.082	0.162
M	639	0	5	4	2	3.585	1.145	0.045	0.089

MTF ages < 6y									
	n	Min age	Max age	Median	iqr	Mean	SD	SE	CI
Gender									
F	3444	0	5	3	2	2.97	1.241	0.021	0.041
M	10,784	0	5	3	2	3.084	1.225	0.012	0.023

### Outside of the Military Treatment Facility

Racial data was not available in the deidentified MHS database for Tricare-covered children diagnosed with autism in their local communities. We identified statistically significant difference in the average ages of autism diagnosis between males and females less than the age of 18 years (p = 4.4e^−08^). Males less than the age of 18 years had an average autism diagnosis age of 10.8 years. Females less than the age of 18 years had an average autism diagnosis age of 11.5 years (Table 2). No significant difference was noted in the ages of autism diagnosis in children less than the age of 6 years. Males less than 6 years of age had an average autism diagnosis age of 3.6 years. Females less than 6 years of age had an average autism diagnosis age of 3.6 years (Table 2).

## Discussion

The age of autism diagnosis in the military pediatric population within the MTF did not reflect disparities historically seen in other reported autism data whereby non-Caucasian children are diagnosed at older ages (Wiggins et al., 2020). The universal coverage of Tricare, a congressionally- mandated universal health care benefit, for the active-duty military families and equal access to the military treatment facility is likely a protective factor in promoting healthcare equity in the military pediatric population. The lower ages of new autism diagnosis seen in non-White older children suggests lessening disparities. However, this may also be indicative of possible late identification of children not appropriately identified previously at younger ages compared to their White counterparts with autism, and therefore diagnosed at slightly younger ages than White children with more subtle autism characteristics. Further studies examining the severity of autism characteristics in this group would be beneficial in this regard.

There was no significant difference in age to diagnosis for females versus males diagnosed with autism within the MTF and in females versus males under the age of 6 years who were diagnosed in the community. Average ages to autism diagnosis in the less-than-6 years groups were below the general population’s average age to autism diagnosis of 4 years as reported by the CDC (National Autism Coordinator, 2023), and was not reflective of historic older ages to autism diagnosis in females compared with males (Loomes et al., 2017).

The average ages of new autism diagnosis for both males and females were lower within the MTF compared with Tricare-covered children diagnosed in the community. Longer wait periods and difficulties accessing care for developmental behavioral concerns outside of the MTF likely explain this disparity. Military children receiving care in MTFs around the world have the added benefit of autism-diagnosing military healthcare providers traveling to remote locations to provide services. This helps to reduce time to autism diagnosis and promotes quicker access to supportive services and therapies.

This study did have limitations. One of the limitations in this study was the large proportion of the study population with an unclear racial identification listed in the study database (racially identified as other, unknown, or N/A). This complicated the interpretation of the impact of race on the age of autism diagnosis in the total military population studied. However, information on racial identity was available for a significant number of individuals (n = 6381) who identified as either American Indian or Alaskan Native, White, Asian, Black or African American, Native Hawaiian or Other Pacific Islander. The percentages of individuals identifying in the various racial groups were found to be commensurate with the percentages of these groups within the larger active-duty military population (2021 Demographics Report). The information collected on age of autism diagnosis among the various racial groups is therefore felt to be an accurate representation.

Another limitation in this study was the lack of sufficient data on socioeconomic status to evaluate its potential impact on age to autism diagnosis within the military. Sponsor’s rank (pay grade) was not used as a proxy for family’s socioeconomic status given insufficient data availability of sponsors’ ranks and poor reliability as rank may not be updated regularly in the database. While access to the MTF is available to active-duty families with Tricare coverage, confounding factors such as availability of reliable transportation, ability of a caregiver to take time off work for evaluations, etc. may limit access to care.

This study highlights the protective factor of universal Tricare coverage and access to the MTF for our military pediatric population on the age of autism diagnosis. Universal health coverage may be an important factor in working to reduce healthcare disparities in autism diagnosis in the general population. This study did not capture potential effects of socioeconomic factors that may limit this access. Future studies are needed to better characterize possible effects of socioeconomics on autism diagnosis and access to care within the military population.

## Data Availability

The data that support the findings of this study are available within the paper. The views expressed are those of the author(s) and do not reflect the official policy of the Department of the Army, the Department of Defense, or the U.S. Government.
